# Control of airborne particles in surgical procedures during the Covid-19 pandemic: scoping review

**DOI:** 10.1590/1980-220X-REEUSP-2021-0579en

**Published:** 2022-07-22

**Authors:** Giovana Caetano de Araújo Laguardia, Vilanice Alves de Araújo Püschel, Patrícia Peres de Oliveira, Luciane Ribeiro de Faria, Ricardo Bezerra Cavalcante, Angélica da Conceição Oliveira Coelho, Kelli Borges dos Santos, Fábio da Costa Carbogim

**Affiliations:** 1 Universidade Federal de Juiz de Fora, Faculdade de Enfermagem, Juiz de Fora, MG, Brazil.; 2 Universidade de São Paulo, Escola de Enfermagem, Departamento de Enfermagem Médico-Cirúrgica, São Paulo, SP, Brazil.; 3 Centro Brasileiro para o Cuidado à Saúde Baseado em Evidências: Centro de Excelência do JBI (JBI Brasil), São Paulo, SP, Brazil.; 4 Universidade Federal de São João del-Rei, Divinópolis, MG, Brazil.

**Keywords:** Surgicenters, Aerosols, Infection Control, Coronavirus Infections, Severe Acute Respiratory Syndrome, Centros Quirúrgicos, Aerosoles, Control de Infecciones, Infecciones por Coronavirus, Síndrome Respiratorio Agudo Grave, Centro Cirúrgico, Aerossóis, Controle de Infecções, Infecções por Coronavírus, Síndrome Respiratória Aguda Grave

## Abstract

**Objective:**

To map the technical and managerial strategies for the management and reduction of airborne particles production in surgical procedures settings during the Covid-19 pandemic.

**Method:**

Scoping review, according to the Joana Briggs Institute methodology, based on documents indexed in MEDLINE, VHL, CINAHL Cochrane, Embase, Scopus, Web of Science, and gray literature, published in Portuguese, English, or Spanish. All studies from indexed scientific journals and recommendations published by international agencies or academic associations from 2019 to January 2022 were considered. Findings were summarized and analyzed using descriptive statistics and narrative synthesis.

**Results:**

Twenty-two studies were selected, 19 of which were published in English, two in Spanish, one in Portuguese, with a predominance of literature reviews. Findings were categorized into recommendations for the environment, the team, and the surgical technique.

**Conclusion:**

The review mapped the technical and managerial strategies for the management and reduction of the airborne particles production in surgical procedures settings. They involve from the use of personal protective equipment, training, anesthetic modality, airway manipulation, to the execution of the surgical technique.

## RESUMO

**Objetivo:**

Mapear as estratégias técnicas e gerenciais para o manejo e a redução da produção de partículas aéreas em ambientes de procedimentos cirúrgicos durante a pandemia da Covid-19.

**Método:**

Revisão de escopo, de acordo com metodologia do Joana Briggs Institute, a partir de documentos indexados nas bases MEDLINE, BVS, CINAHL Cochrane, Embase, Scopus, Web of Science e literatura cinza, publicados em português, inglês ou espanhol. Foram considerados todos os estudos provenientes de periódicos científicos indexados e recomendações publicadas por órgãos internacionais ou associações acadêmicas, de 2019 a janeiro de 2022. Os achados foram sumarizados e analisados por estatística descritiva e síntese narrativa.

**Resultados:**

Foram selecionados 22 estudos, sendo 19 publicados em inglês, dois em espanhol, um em português, com predominância de revisões da literatura. Os achados foram categorizados em recomendações para o ambiente, a equipe e a técnica cirúrgica.

**Conclusão:**

a revisão mapeou as estratégias técnicas e gerenciais para o manejo e a redução da produção de partículas aéreas nos ambientes de procedimentos cirúrgicos. Envolvem desde o uso de equipamentos de proteção individual, treinamentos, modalidade anestésica, manipulação de vias aéreas, até a execução da técnica cirúrgica.

## RESUMEN

**Objetivo:**

mapeo de las estrategias técnicas y de gestión para el manejo y la reducción de la producción de partículas aéreas en ambientes de procedimientos quirúrgicos durante la pandemia de Covid-19.

**Método:**

revisión de alcance, de acuerdo con metodología JBI, con base en documentos indexados en las bases MEDLINE, BVS, CINAHL Cochrane, Embase, Scopus, Web of Science y literatura gris, publicados en portugués, inglés o español. Se consideraron todos los estudios provenientes de periódicos científicos indexados y recomendaciones publicadas por órganos internacionales o asociaciones académicas, de 2019 a enero de 2022. Los hallazgos fueron sintetizados y analizados por estadística descriptiva y síntesis narrativa.

**Resultados:**

Se seleccionaron 22 estudios, siendo que 19 fueron publicados en inglés, dos en español, uno en portugués, con predominancia de revisiones de literatura. Los hallazgos fueron categorizados en recomendaciones para: el ambiente; el equipo y la técnica quirúrgica.

**Conclusión:**

la revisión mapeó las estrategias técnicas y de gestión para el manejo y la reducción de la producción de partículas aéreas en los ambientes de procedimientos quirúrgicos. Comprenden desde el uso de equipo de protección individual, entrenamientos, modalidad anestésica, manipulación de vías aéreas, incluso la ejecución de la técnica cirúrgica.

## INTRODUCTION

Severe acute respiratory syndrome caused by coronavirus 2 (SARS-CoV-2), better known as COVID-19, represents one of the greatest challenges for global public health^(1)^. Since its identification in December 2019 in the Chinese province of Wuhan, COVID-19 was responsible for thousands of deaths in several countries^(2)^. As the disease progresses, on March 11, 2020, the World Health Organization (WHO) declared COVID-19 a pandemic and emerging disease^(2)^. However, the knowledge under construction about the pathogenicity of the virus and its ability to mutate has required rapid responses from health systems, grounded on decision-making based on the best scientific evidence^(3)^.

SARS-CoV-2 is a respiratory virus that initially settles in the upper respiratory tract and can be transmitted by airborne particles such as droplets and aerosols. Droplets are macroparticles that reach up to one meter away after being expelled, while aerosols are microparticles that remain suspended in the environment for a long period and can be transported through the air, increasing the transmission potential^(4)^.

Given this scenario and the need to protect health teams and patients, precautionary measures were required and have been constantly reassessed^(5–7)^. More specifically, in the operating room environment, elective surgeries were initially suspended until a more favorable epidemiological scenario was reached^(6)^. These measures were necessary due to the high risk of exposure that the procedures performed in the operating room pose to the healthcare team and patients regarding SARS-CoV-2 infection^(7)^.

Among the procedures with the greatest potential to produce aerosol, intubation/extubation, manual airway ventilation, the use of electrocautery and high-speed drills stand out^(8)^. Recent studies have been conducted to estimate the concentration of dispersed particles during surgical procedures, aiming at increasing the understanding of the possible risks of exposure to SARS-CoV-2 during surgeries^(8,9,10,11)^.

Researchers quantified the average concentration of particles using an optical meter during endonasal surgeries. They found that close to the surgeon there was an increase in the average concentration of 2,445 particles/cubic feet during the use of the drill and 1,825 particles/cubic feet during the use of a microdebrider^(11)^. Although associated with a surgical modality, these data reinforce the need to adopt measures that are known to be effective for protection and prevention of infection, such as the correct use of personal protective equipment (PPE)^(11,12)^. It should be noted that, besides the use of PPE, studies indicate measures related to controlling the amount of inoculum in the environment, as well as environmental conditions such as temperature and humidity, which can change the viability time of aerosolized viral particles^(11,12,13,14)^.

Despite the advancement of knowledge on the prevention and control of COVID-19, the literature still lacks evidence and mapping of comprehensive recommendations related to measures to control the production of airborne particles in surgical procedures settings. Therefore, a scoping review is warranted, to map the technical and managerial strategies for the management and reduction of the production of airborne particles in surgical procedures settings during the Covid-19 pandemic. A preliminary search was performed in PROSPERO, MEDLINE, Cochrane Database of Systematic Reviews and JBI Evidence Synthesis and no reviews with this approach, completed or in progress, were identified.

Given what has been said, the study aims at mapping the technical and managerial strategies for the management and reduction of the production of airborne particles in surgical procedures settings during the Covid-19 pandemic.

## METHOD

### Design of Study

This is a scoping review, guided by the JBI review methodology^(15)^. This methodology allows mapping concepts, clarifying areas of knowledge and possible gaps. To conduct the study, five steps were followed^(15)^: identification of the research question; survey of relevant studies, considering the scope and coverage of the review; selection of studies, according to predefined criteria; data mapping; and presentation of results. The recommendations of the Prisma Extension for Scoping Reviews (Primas-ScR) checklist were also considered^(16)^.

The review was registered on the platform *Open Science Framework*, with identification DOI 10.17605/OSF.IO/4AW57.

### Guiding Question, Search, and Inclusion Criteria

The study guiding question was: what are the technical and managerial strategies for the management and reduction of the production of airborne particles in surgical procedures settings during the Covid-19 pandemic?

The studies included in this scoping review were selected using the PCC (Population, Concept and Context) mnemonic strategy, as follows: population (P), patients aged 18 years or older; concept (C), technical and managerial strategies used to manage and reduce the spread of airborne particles in surgical procedures settings; context (C), operating room during the Covid-19 pandemic. Technical strategies are understood as the set of assistance procedures, adjusted to control the production of airborne particles. Management strategies, on the other hand, refer to a set of actions involving planning and evaluation aimed to control the production of airborne particles.

For the review, documents were included, such as scientific articles, theses, dissertations, books, protocols, and recommendations on technical and managerial strategies used for the management and reduction of the spread of airborne particles in surgical procedures settings for patients over 18 years of age. Moreover, documents should have been published from 2019, year of first notification of the disease, in English, Portuguese and Spanish.

Letters to the editor, abstracts in events annals, research protocols, and documents in the field of dentistry were excluded.

**Chart 1.**
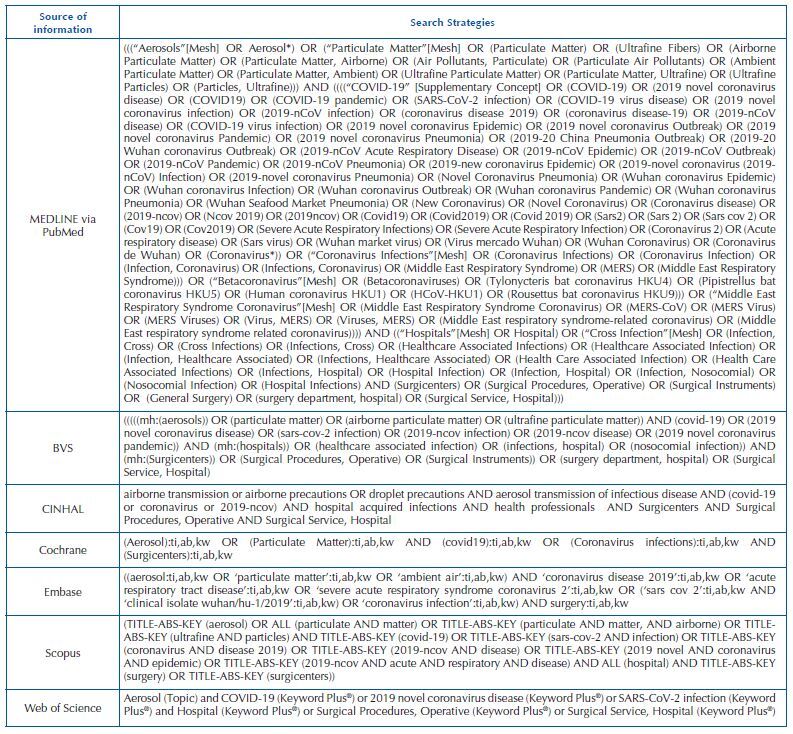
Search strategy for document retrieval – Juiz de Fora, MG, Brazil, 2022.

To search and identify the documents/studies, the following electronic sources were used: *Medical Literature Analysis and Retrieval System Online* (MEDLINE) via PubMed, Virtual Health Library (VHL), *Cumulative Index to Nursing and Allied Health Literature* (CINAHL), *Cochrane Library*, Embase, Scopus, and *Web of Science*. Access to the full texts was made through the Portal of Periodicals of the Coordination for the Improvement of Higher Education Personnel (CAPES), with use of *proxy* from the Universidade Federal de Juiz de Fora (UFJF). As a search strategy for studies/documents, the structuring presented in Chart 1 was used.

To identify other relevant studies/documents in the gray literature, the following sources were searched: *Google Scholar (first five pages); Brazil – Portal of Theses and Dissertations for CAPES; Search system of the National Health Surveillance Agency (Anvisa); United States of America (USA) – Search engine American College of Surgeons (ACS); search system for Centers for Disease Control and Prevention (CDC); European continent -System for Information on Gray Literature in Europe (Opengrey); UK – British Library EThOS; Sweden and other Scandinavian countries – Academic Archive Online (DIVA) and Australia and New Zealand – National Library of Australia (Trove).* For the search in the gray literature, combinations of the keywords “*Surgical Procedures*”; “*Covid-19*” and “*aerosol*” were used.

The searches took place on July 16, 2021, with a new search being established, in all bases and sources, on January 23, 2022.

### Data Selection, Analysis and Treatment

Following the databases and sources search, the documents were selected based on the research question. The results obtained were exported to the reference manager Rayyan^***®***^, developed by *Qatar Computing Research Institute* (QCRI). The manager allowed the removal of duplicate documents, the independent selection and screening of documents by two reviewers. Therefore, the first phase was the reading of titles and abstracts, independently and blindly, by the two reviewers. Disagreements were resolved by discussion between the two reviewers and, when necessary, the participation of a third reviewer. For documents meeting the inclusion criteria, the second phase was carried out, involving the reading of the documents in their entirety, seeking information about technical and managerial strategies for the management and reduction of the production of airborne particles in surgical procedures environments during the Covid-19 pandemic. Disagreements were resolved with the participation of a third researcher.

The information from the documents selected for analysis was independently extracted by two reviewers, using spreadsheets from Microsoft Excel^***®***^. A third reviewer participated in the validation of the information and in the discussion to establish consensus among the authors, when required. The mapping of information was established based on the JBI instrument to characterize the productions^(15)^. For data extraction, a chart was created that included authorship, year of publication, language and country of origin, type of study and objectives, surgical procedure and technical/managerial strategies for the management and reduction of the spread of airborne particles in surgical procedures settings.

Subsequently, data were categorized into recommendations, according to the technical and managerial strategies for the management and reduction of airborne particles in the surgical environment.

Based on the categorized data, a narrative presentation of the information was performed.

## RESULTS

**Figure 1.**
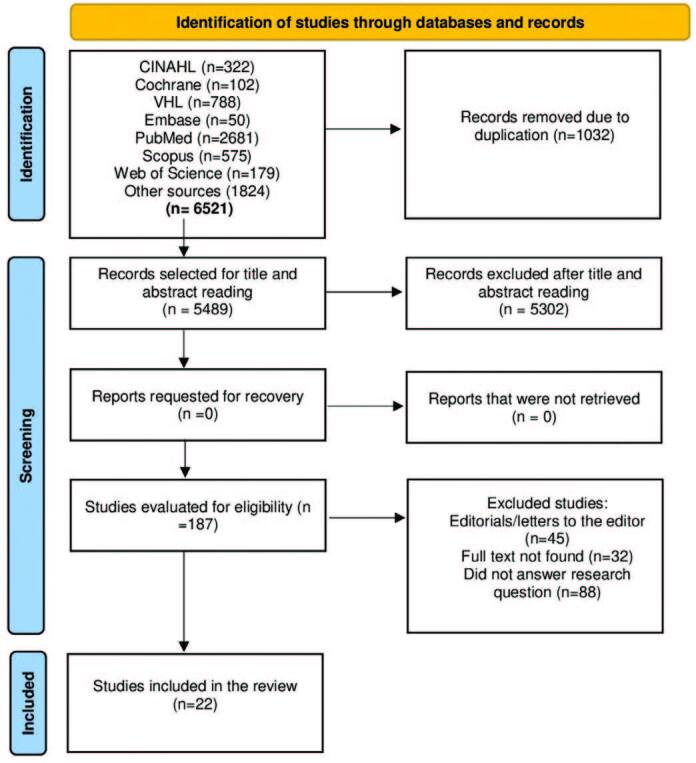
PRISMA-ScR flowchart for the selection of publications^(16)^ – Juiz de Fora, MG, Brazil, 2022.

The search in the investigation bases retrieved 6,521 potentially relevant documents/studies. A total of 1,032 duplicate documents were excluded. A total of 5,489 publications were analyzed by title and abstract, and 5,302 documents/studies were excluded because they did not meet the inclusion criteria. Thus, 187 documents/studies were fully evaluated for eligibility. At the end, 22 documents/studies^(12,17–37)^ were included to compose the final review sample (Figure 1).

**Chart 2.**
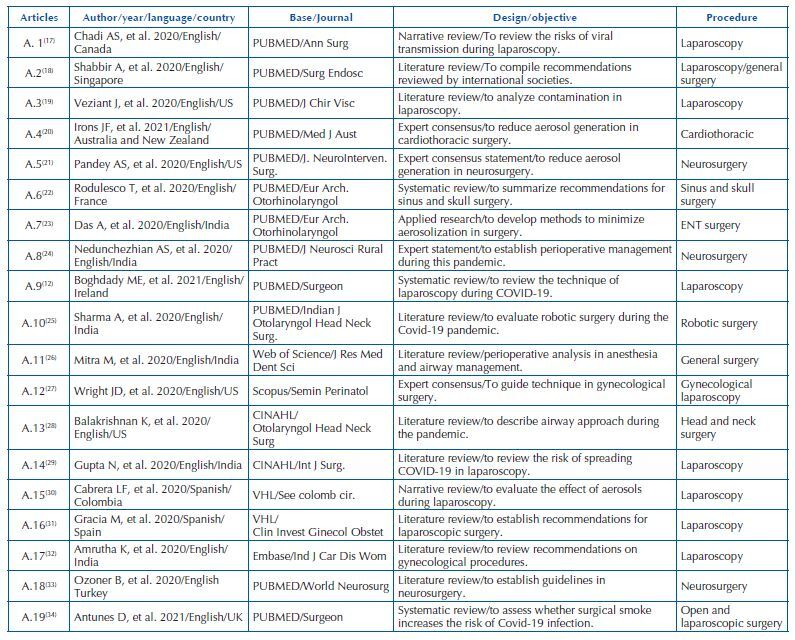
Characterization of the articles included in the review – Juiz de Fora, MG, Brazil, 2022.

**Chart 3.**
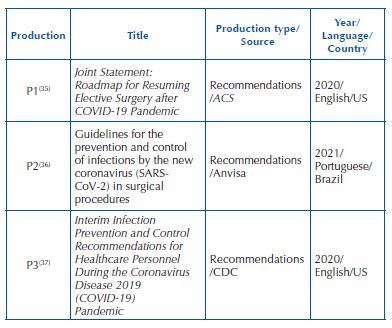
Characterization of publications retrieved by searching the gray literature – Juiz de Fora, MG, Brazil, 2022.

Of the 22 documents/studies included, 19 were published in English^(12,17–29,32–35,37)^, two in Spanish^(30,31)^ and one in Portuguese^(36)^. As for the origin, nine were produced in the American continent^(17,19,21,27,28,30,35–37)^, seven in the Asian continent^(18,23–26,29,32)^, five on the European or Eurasian continent^(12,22,31,33,34)^, and one in Oceania^(20)^. Among the studies, 14 were reviews^(12,17–19,22,25,26,28–34)^, four were expert consensus statements^(20,21,24,27)^, three were protocol recommendations^(35–37)^, and one was related to the development of a technique for aerosol reduction^(23)^. The characterization of the articles included is shown in Chart 2 and that of the gray literature publications is shown in Chart 3.

**Chart 4.**
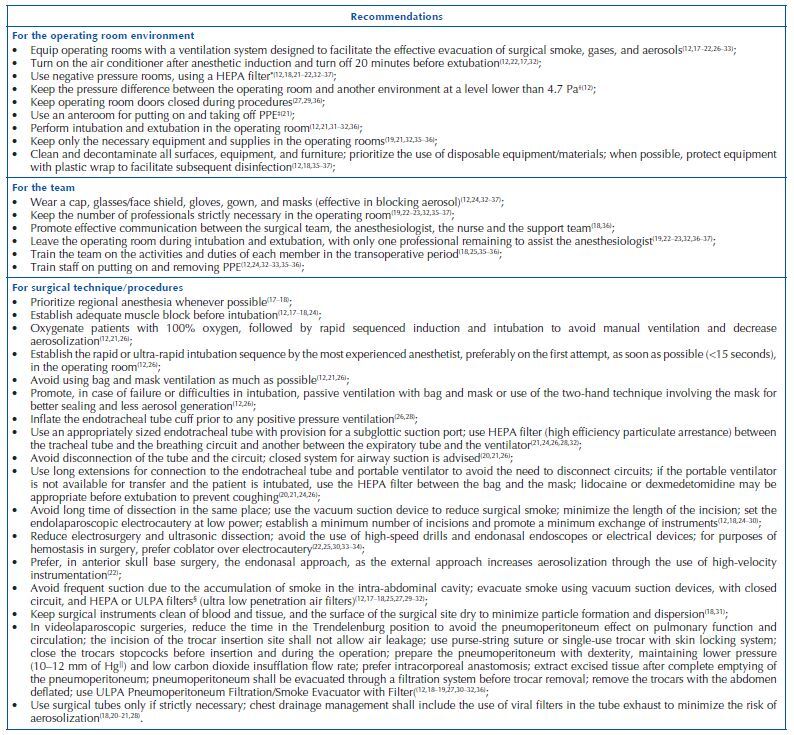
Main technical and managerial recommendations to reduce the production of airborne particles in surgical procedures settings during the Covid-19 pandemic – Juiz de Fora, MG, Brazil, 2021.

The information in the documents/studies included evidenced three themes with technical and managerial recommendations to reduce the production of airborne particles in surgical procedures settings during the Covid-19 pandemic: recommendations for the environment; recommendations for the team; and recommendations for the surgical technique/procedure (Chart 4).

*High efficiency particulate arrestance filter is a filter with high efficiency in the separation of particles; ^†^Atmospheric pressure in Pascal (equivalent to 4.x10^–5^ atmosphere); ^‡^Individual protection equipment; ^§^Ultra low penetration air filter, a filter capable of removing 99.999% of particles from the air; ^||^Unit of pressure in millimeters of mercury.

## DISCUSSION

The studies included in this review were mostly literature reviews^(12,17–19,22,25,26,28–34)^, produced mainly by authors from the Asian continent^(18.23–26,29,32)^. Rapid reviews prevailed, focusing on compiling recommendations from experts and international bodies aiming at minimizing perioperative aerosol production. These reviews, produced mostly in 2020, are consistent with the initial situation of the pandemic that requires speed in the establishment of protocol behaviors and recommendations for health services. Regarding the Asian continent, it is inferred that this predominance is related to the search for recommendations in the first continent to notify and initiate measures to contain the spread of Covid-19 in surgical centers^(34)^.

As for surgical procedures, the highest frequency in the guidelines was focused on videolaparoscopic surgery^(12,17–19,27,29–32)^. It is a minimally invasive surgical approach that uses high-resolution cameras and appropriate instruments inserted through trocars in small incisions^(12,19)^. This technique allows a closed approach to the surgical site; however, there is high chance of particles scattering along with the smoke from electrical or ultrasonic equipment^(12)^.

Regardless of the surgical technique, scientific societies and world health agencies initially recommended postponing elective surgeries, except in regions with a favorable epidemiological situation^(2,38,39)^. However, with the sedimentation of knowledge about the disease and the mass vaccination of the world population, even if in a heterogeneous way, the surgeries that were once postponed are being resumed^(34)^. Therefore, even for vaccinated patients, screening and complementary tests prior to surgeries are important^(34,36,37)^. These behaviors increase safety for patients and the healthcare team^(34)^.

Therefore, investigations^(40–42)^ first recommend patients screening, with anamnesis aimed at identifying signs and symptoms of Covid-19. In addition, they also recommend carrying out molecular or immunological diagnostic tests and, when not available in a timely manner, considering the patient as a possible carrier of Covid-19^(41,42)^. A study^(33)^ also recommends chest computed tomography as an additional possibility for patient screening.

Regarding the recommendations for the operating room settings, there is an indication of an exclusive operating room and post-anesthetic recovery room for patients suspected or diagnosed with Covid-19^(43,44)^. It is also important to establish a circulation flow and equip operating rooms with a ventilation and filtration system, to favor the safe elimination of smoke, gases, and aerosols^(25,43)^. Authors^(12,22)^ highlight the importance of operating rooms equipped with high-efficiency filters, which guarantee about 25 filtrations per hour and with a negative pressure of at least −4.7 Pa in relation to the antechamber. If these resources cannot be used, the maintenance of a stable pressure should be encouraged. Therefore, it is recommended to turn off the air conditioning equipment during aerosol generating procedures^(32)^. Care with filtration and pressure shall be maintained during the process of operating rooms final cleaning^(12,22,32)^.

The multidisciplinary surgical team shall undergo specific training on flows, disposal of contaminating materials, biological risk, with emphasis on protection through the use of PPE^(25,43)^. The use of PPE such as respiratory protection masks for droplets and aerosols, caps, glasses/face shield, gloves, gown, and waterproof footwear is essential to preserve the teams’ health^(17–20)^. The PPE guidelines shall also clarify about donning and doffing, hand hygiene before and after equipment removal, which equipment shall be discarded or reused, as well as the orderly flow for this process^(17,18,43)^.

A study highlights the importance of carrying out briefings among team members to assign roles, discuss surgery, identify aerosol-generating procedures, and review recommendations^(20)^.

As for the recommendations on surgical procedures, these range from the selection of the anesthetic modality, the adequate manipulation of the airways, to the execution of the surgical technique^(12,20,21,24)^. In the context of the Covid-19 pandemic, the main objective is to reduce the production and dispersion of air particles as much as possible, opting for procedures that do not produce aerosols, gases or fumes^(20,45)^. Whenever possible, anesthesia through locoregional blocks should be used, considering that general anesthesia requires manipulation of the pathways, with ventilation maneuvers, tracheal intubation and, consequently, aerosol production^(17,18)^. However, when tracheal intubation is necessary, it shall be performed by an experienced professional, in the shortest possible time and with a limited number of people present^(20,26)^. Research recommends that other team professionals only enter the operating room after an average interval of 10 minutes, which guarantees at least four cycles of ambient air filtration^(18,22)^.

With regard to the surgical modality, i.e., minimally invasive or open surgery, there are no clear recommendations in the literature on which technique produces fewer airborne particles^(40–43)^. The included literature identifies thoracic, neurological, otorhinolaryngological, maxillofacial, and laparoscopic surgeries as procedures related to the high production of air particles^(20,21,29)^. Among them, research has reported a greater risk related to laparoscopy, due to gas leakage from the pneumoperitoneum, which can contain high concentrations of suspended virus^(12,43)^. In this regard, the safe management of pneumoperitoneum is recommended, with low pressures of carbon dioxide and the use of a suction and frequent filtration system to avoid the accumulation of surgical smoke (plume) in the abdominal cavity^(18)^.

Another recommendation identified in the studies concerns the size and number of surgical incisions, with the risk of producing surgical smoke being proportional to the size and number of incisions^(17,18)^. In addition, all energy-generating equipment, such as electrocautery or ultrasonics, shall be set to low power to reduce the production of aerosolized particulate matter^(18,30,32)^.

For surgical completion, the authors recommend the use of tubes only if strictly necessary and the synthesis with absorbable threads or any closure device that reduces gases leakage through the surgical wound^(18,29,33)^.

The literature still lacks further studies to determine if there is a direct relationship between the transmission of Covid-19 and surgical smoke^(34)^. Thus, it is up to government health agencies, responsible for guidelines, to monitor the production of evidence syntheses, adjusting or modifying the recommendations, when necessary.

This investigation has as limitations the inclusion of studies in only three languages and the time frame. The former limitation is related to the technical capacity of the team and the lack of reliable resources for the translation of studies into other languages. As for the latter limitation, despite being linked to Covid-19, a recently emerging disease, it may have been a limiting factor for the mapping of recommendations in other pandemic contexts.

It is believed that the results of the present investigation will be able to provide a set of actions for settings of surgical procedures performed during the COVID-19 pandemic and in other epidemic scenarios.

## CONCLUSION

The mapping of strategies for managing the production of airborne particles in surgical rooms during the Covid-19 pandemic identified technical and managerial recommendations regarding the operating room environment, the multiprofessional team, and the surgical procedures themselves.

Technical strategies are mainly related to wearing complete attire, recommending regional anesthesia when possible, avoiding manual bag and mask ventilation, prioritizing rapid sequence intubation, minimizing the length and number of surgical incisions, to reduce electrosurgery, to use ultrasonic dissection, installation of tubes and, in video surgeries, to use techniques that reduce the accumulation or extravasation of gas or surgical smoke. Management strategies are related to training the multidisciplinary team, controlling the movement of people, providing equipment and supplies that are strictly necessary for the procedures and using rooms with a ventilation system and negative pressure.

The results presented are intended to support safe clinical practice and collaborate with new research on airborne particle control strategies in surgical procedure settings. However, the results of this review are provisional and may change as scientific discoveries about Covid-19 advance. Thus, new studies are recommended that include research with a high level of evidence, produced over the time frame of the Covid-19 pandemic.
